# An invasive insect herbivore disrupts plant volatile-mediated tritrophic signalling

**DOI:** 10.1007/s10340-017-0877-5

**Published:** 2017-05-13

**Authors:** Letizia Martorana, Maria Cristina Foti, Gabriele Rondoni, Eric Conti, Stefano Colazza, Ezio Peri

**Affiliations:** 1 Dipartimento di Scienze Agrarie, Alimentari e Forestali, Università degli Studi di Palermo, Viale delle Scienze, 90128 Palermo, Italy; 2 Dipartimento di Scienze Agrarie Alimentari e Ambientali, Università degli Studi di Perugia, Borgo XX Giugno 74, 06121 Perugia, Italy

**Keywords:** Oviposition-induced plant volatiles, *Halyomorpha halys*, Insect invasion, Multiple attack, *Trissolcus basalis*

## Abstract

Plants respond to insect attack by emission of volatile organic compounds, which recruit natural enemies of the attacking herbivore, constituting an indirect plant defence strategy. In this context, the egg parasitoid *Trissolcus basalis* is attracted by oviposition-induced plant volatiles emitted by *Vicia faba* plants as a consequence of feeding and oviposition by the pentatomid host *Nezara viridula.* However, this local tritrophic web could be affected by the recent invasion by the alien pentatomid bug *Halyomorpha halys*, an herbivore that shares the same environments as native pentatomid pests. Therefore, we investigated in laboratory conditions the possible impact of *H. halys* on the plant volatile-mediated signalling in the local tritrophic web *V. faba*–*N. viridula*–*T. basalis*. We found that *T. basalis* wasps were not attracted by volatiles induced in the plants by feeding and oviposition activities of *H. halys,* indicating specificity in the wasps’ response. However, the parasitoid attraction towards plant volatiles emitted as a consequence of feeding and oviposition by the associated host was disrupted when host, *N. viridula*, and non-associated host, *H. halys*, were concurrently present on the same plant, indicating that invasion by the alien herbivore interferes with established semiochemical webs. These outcomes are discussed in a context of multiple herbivory by evaluating the possible influences of alien insects on local parasitoid foraging behaviour.

## Key Message

- We investigated the attraction of the indigenous egg parasitoid *Trissolcus basalis* towards plant volatiles induced by *Nezara viridula* versus alien *Halyomorpha halys*.
- The parasitoid was attracted by plant volatiles induced by *N. viridula*, whereas it was not attracted by volatiles induced by *H. halys*.
- The parasitoid attraction towards plant volatiles induced by associated host was disrupted when associated host and non-associated host were concurrently present on the same plant, indicating that invasion by the alien herbivore interferes with the established semiochemical interaction.

## Introduction

The reproductive success of insect parasitoids and their efficacy in controlling herbivorous insect pest populations in biological control programmes is closely related to their ability to locate hosts at the suitable stage (Pickett and Khan 2016; Kaiser et al. 2016). In field conditions, the in-flight host-searching ability of insect parasitoids is largely based on chemical cues, among which the volatile organic compounds (VOCs) emitted by plants in response to herbivorous insect activities play a central role (Dicke 2016; Kaiser et al. 2017). It is now well established that the main insect activities that induce VOCs are feeding (herbivore-induced plant volatiles, HIPVs) or egg deposition (oviposition-induced plant volatiles, OIPVs) (Hilker and Fatouros 2015; Pashalidou et al. 2015). From the plant perspective, the emission of VOCs that recruit natural enemies represents an indirect defence strategy, as these volatiles do not have direct impact on the attacking herbivore. Specifically, OIPVs are able to attract egg parasitoids that might improve plant fitness, as the herbivorous insect can be killed before plant damage occurs (Pierik et al. 2014; Fatouros et al. 2016). In a natural environment, plants are subject to stress by various herbivorous insects and, as a result, the plant’s phenotype changes significantly reflecting differences in blends of the volatiles they produce (Moayeri et al. 2007; Dicke 2016). Alteration of induced volatile blends in plants under multiple and simultaneous herbivore attack may depend, among several factors, on the insect feeding habits (chewing, piercing or sucking), the plant organ attacked (above- or belowground) or the strength of the herbivore damage (herbivore density, timing and location of the different attackers) (De Rijk et al. 2013; Ponzio et al. 2014; Kroes et al. 2015). In this context, the colonization of a new environment by an alien herbivore could interfere with the plant indirect defences due to the lack of plant–herbivore coevolution (Desurmont et al. 2014). The attack of alien herbivores could influence parasitoid foraging behaviour in different ways, for example, through direct attraction of parasitoids towards infested plants, or modification of normal attraction towards plants with concurrent infestation with local host herbivore. Studies focused on multiple attacks are rather limited and most consider only endemic herbivores.

The females of the egg parasitoid *Trissolcus basalis* (Wollaston) (Hymenoptera: Platygastridae) are attracted by OIPVs emitted by *Vicia faba* L. plants as a consequence of feeding and oviposition by *Nezara viridula* (L.) (Heteroptera: Pentatomidae) (Colazza et al. 2004a, b). Recent observations demonstrate that this infochemical network is affected by biotic and abiotic stress. In fact, the ability of *T. basalis* females to exploit *V. faba* OIPVs is disrupted when the plants are simultaneously infested by *N. viridula* and the non-host, *Sitona lineatus* (L.) (Coleoptera: Curculionidae) (Moujahed et al. 2014), or is enhanced when *N. viridula* attack plants that are under water stress conditions (Salerno et al. 2017).

The brown marmorated stink bug, *Halyomorpha halys* (Stål) (Heteroptera: Pentatomidae), native of east Asia (Lee et al. 2013), is a very polyphagous herbivore of over 100 host plants, including agricultural, horticultural and ornamental plants (Leskey et al. 2012; Haye et al. 2014). After its introduction to North America and Europe, *H. halys* rapidly spread becoming a harmful invasive species that can cause severe economic losses in orchards and field crops (Rice et al. 2014; Haye et al. 2015). In Italy, *H. halys* was detected for the first time in September 2012 in the province of Modena, and to date, its presence is limited in the northern regions where it causes damages mainly in nectarine orchards (Cesari et al. 2015; Roversi et al. 2016); nonetheless, it is expected to spread widely and to increase its importance as crop pest. From an ecological point of view, *H. halys* has the potential to share the same local community structures of local pentatomid pests adding a resource to the environment for their indigenous parasitoids. Therefore, the presence of invasive *H. halys* could determine important ecological consequences for plant–pentatomid herbivore–parasitoid interactions, depending on the parasitoid’s ability to recognize the herbivore and successfully develop in *H. halys* eggs. In this view, the deeply investigated interactions among *V. faba*–*N. viridula*–*T. basalis* could represent a model system for evaluating the ecological effects of the of invasive *H. halys* on local tritrophic web. Moreover, preliminary observations under laboratory conditions showed that *T. basalis* is able to reproduce in *H. halys* eggs albeit with very low rates of parasitism (M.C. Foti, personal observations). Therefore, the wasp’s ability to complete host selection sequence could determine the development of a ‘new association’ between *T. basalis* wasps and the potential new host *H. halys*.

In the present work, laboratory experiments were conducted to determine the impact of *H. halys* on the plant volatile-mediated signalling in the local tritrophic web *V. faba*–*N. viridula*–*T. basalis*. The impact of *H. halys* was addressed both as a single stress factor, to investigate whether *T. basalis* females were attracted to *V. faba* plants infested by *H. halys,* and as a concurrent infestation by *H. halys* and *N. viridula*, to investigate whether the activity of the alien *H. halys* disrupts the attraction of parasitoid females towards volatiles emitted by *V. faba*-damaged plants.

## Materials and methods

### Plants

Seeds of broad bean plants (*V. faba* cv. Superaguadulce) were immersed for 24 h in a slurry of water and soil (1:4) to start germination and then individually planted in plastic pots (9 × 9 × 13 cm) filled with a mixture (1:1) of agriperlite (Superlite, Gyproc Saint-Gobain, PPC Italia, Italy) and vermiculite (Silver, Gyproc Saint-Gobain, PPC Italia, Italy). Plants were grown in a climate-controlled chamber (24 ± 2 °C, 45 ± 10% RH, 12 h:12 h *L*:*D*), watered daily and, from 1 week post-germination, fertilized with an aqueous solution (1.4 g/l) of fertilizer (5–15–45, N–P–K, Plantfol, Valagro, Italy). For the treatments, 3-week-old broad bean plants, with approximately seven fully expanded leaves, were used.

### Insect rearing

The colonies of *N. viridula* and *H. halys* were established from field collected materials around Palermo and Perugia, Italy, and reared separately in insect rearing cages (47.5 × 47.5 × 47.5 cm) (Bug-Dorm-44545, MegaView Science Co. Ltd., Taichung, Taiwan) under controlled conditions (24 ± 2 °C; 70 ± 5% RH; 16 h:8 h *L*:*D*). Both colonies were fed with tomato and/or broad bean plants, fresh organic vegetables, and soybean and sunflower seeds. Food was renewed every 2 days, and water was provided with soaked cotton wool in small containers. Egg masses were collected daily and used to maintain colonies. Stink bugs used in the experiments were from the 1st to the 5th laboratory generations.

The colony of *T. basalis* was originally established from wasps emerging from *N. viridula* sentinel egg masses located in fields around Palermo, Italy, and was reared on *N. viridula* egg masses. Wasps were maintained in 85-ml glass tubes, fed with a honey–water solution (80:20 v/v) and kept in a controlled environment room (24 ± 2 °C; 70 ± 5% RH; 16 h:8 h *L*:*D*). After emergence, male and female wasps were kept together to allow mating. For all bioassays, wasp females, from the 1st to the 7th laboratory generation, 3–5 days old and naïve, were individually isolated in small vials for 12 h and then transferred to the bioassay room to be acclimatized around 1 h before the tests.

### Plant treatments

Potted broad bean plants were exposed to one stink bug female, caged for 24 h on the abaxial surface of an expanded leaf using a clip-cage, which consists in two modified plastic Petri dishes (*Ø* = 3.5 cm; h = 1 cm) with a mesh-covered hole (*Ø* = 3 cm) and the rim covered by a small sponge ring. In these conditions, the stink bugs were allowed to feed and oviposit (exposed plants). The egg masses laid by *H. halys* on the exposed plants ranged from 25 to 30 eggs (*N* = 10, weight average 0.043 ± 0.001 g), while those laid by *N. viridula* ranged from 65 to 75 eggs (*N* = 10, weight average 0.040 ± 0.001 g). Treated plants with empty clip-cage, kept on a leaf for 24 h, were used as control (unexposed plants). At the end of the treatments, the stink bugs and the clip-cages were removed, and after 24 h, the plants were bioassayed according to different combinations of treatment versus control. All the treatments were performed using 10- to 20-day-old stink bug adult females, fed and mated.

To determine the response of *T. basalis* to constitutive volatiles of *V. faba* plants, unexposed plants were tested versus air. To determine the response of *T. basalis* to volatiles induced by plants damaged by *H. halys*, the following combinations were performed:

- Plants exposed to *H. halys* feeding versus unexposed plants
- Plants exposed to *H. halys* feeding and oviposition versus unexposed plants

To determine the response of *T. basalis* to volatiles induced by plants subjected to concurrent infestation of *H. halys* and *N. viridula*, the treatments consisted of:

- Plants exposed to *N. viridula* feeding and oviposition versus unexposed plants
- Plants exposed to *H. halys* feeding and *N. viridula* feeding and oviposition versus unexposed plants
- Plants exposed to *H. halys* feeding and oviposition and *N. viridula* feeding versus unexposed plants
- Plants exposed to *H. halys* feeding and *N. viridula* feeding and oviposition versus plants exposed to *N. viridula* feeding and oviposition.

### Y-tube olfactometer bioassays

Wasp responses to the treated plants were investigated with a dual-choice Y-tube olfactometer made from a polycarbonate body (stem 9 cm; arms 8 cm at 130° angle; ID 1.5 cm) sandwiched between two glass plates. A stream of medical-grade compressed air (approximately 80:20, N_2_:O_2_) coming straight from the cylinder, humidified by bubbling through a water jar, was regulated in each arm by a flow-meter at about 0.5 l min^−1^. The device was illuminated from above by two 22-W cool white fluorescent tubes (full spectrum 5900 K, 11 W; Lival, Italy) and from below by an infrared source (homogeneous emission of wavelengths at 950 nm provided by 108 LEDs). Before entering in the olfactometer arms, each air stream passed through a cylindrical glass chamber (*Ø* = 12 cm; *h* = 52 cm) containing a treated plants odour source. The stimuli were randomly assigned at the beginning of the bioassays and were reversed after testing three parasitoid females. At every switch, the whole system was changed with cleaned parts. At the end of the bioassays, the polycarbonate olfactometer and all glass parts were cleaned with fragrance-free soap, rinsed with demineralised water and dried. The glass parts were then baked overnight at 180 °C. Wasp females were singly introduced into the Y-tube olfactometer, and their behaviour was recorded for 10 min using a monochrome CCD video camera (Sony SSC M370 CE) fitted with a 12.5–75 mm/F 1.8 zoom lens. The camera lens was covered with an infrared pass filter (Kodak Wratten filter 87 Å) to remove visible wavelengths. Analogue video signals from the camera were digitized by a video frame grabber (Studio PCTV–Pinnacle Systems, Mountain View, CA). Digitized data were processed by XBug, a video tracking and motion analysis software as described in Colazza et al. (1999).

Wasp response was measured in terms of residence time, i.e. the time spent by the wasps in each arm during the bioassay. The Y-tube olfactometer bioassays were carried out as paired choices, in which the test odour sources were always tested versus a control odour as detailed above. Bioassays were conducted from 10:00 to 13:00 h under controlled conditions (26 ± 1 °C; 50 ± 5% RH).

### Statistical analysis

For the bioassays, the time spent by wasp females in each arm was statistically compared by parametric paired *t* tests for dependent samples. The time spent by the wasps in the common arm was excluded from the analysis. Data were analysed using the STATISTICA 7 software (StatSoft 2001).

## Results

Response of *T. basalis* to volatiles induced by plants damaged by *H. halys.*

**Fig. 1:**
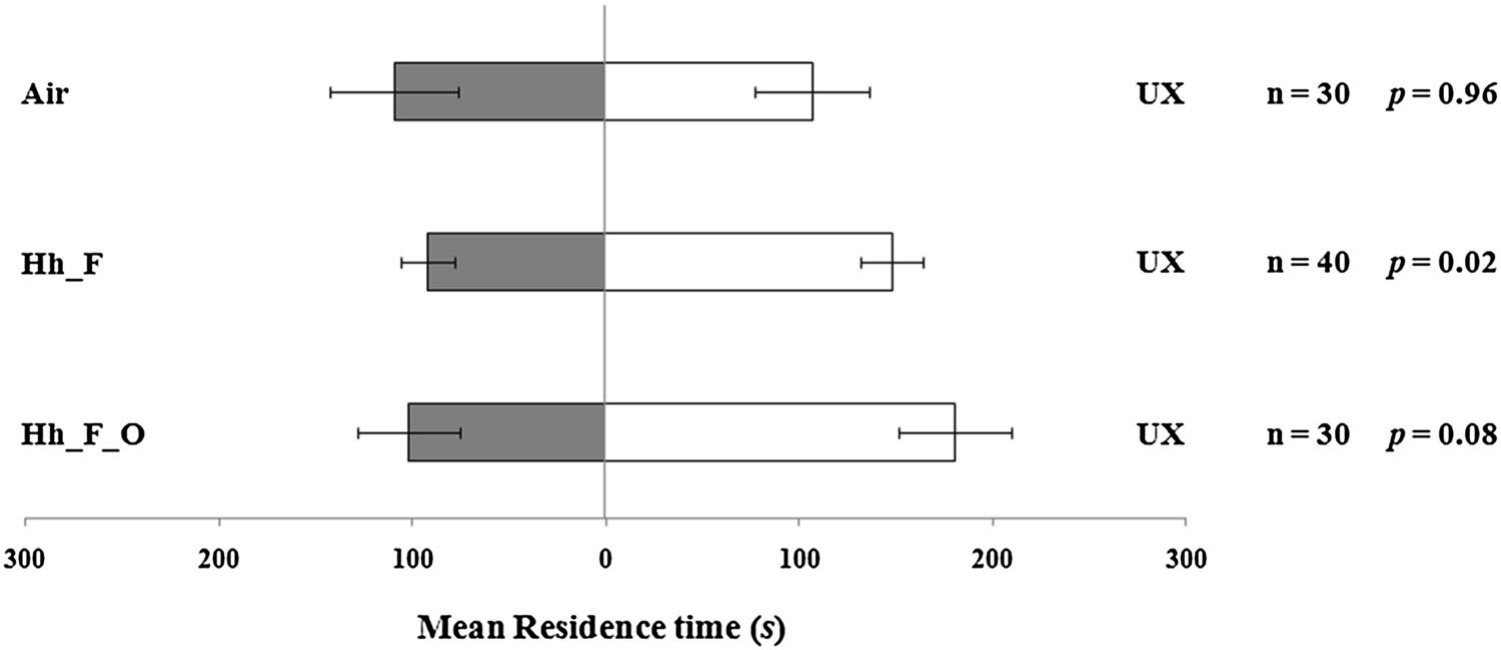
Response of *Trissolcus basalis* females to *Vicia faba* plant volatiles induced by *Halyomorpha halys*. Plant treatments: *H. halys* feeding and oviposition (Hh_F_O); *H. halys* feeding (Hh_F); unexposed (UX). *n* = number of replicates. *Bars* represent mean (±SE) of the time spent by wasp females in each arm of the Y-tube olfactometer over an observation period of 300 s (paired *t* tests)

*Trissolcus basalis* females were not attracted to unexposed plants when tested versus air (*t* = −0.05; *df* = 29; *p* = 0.96) (Fig. 1). Wasp females were not attracted to VOCs emitted by *V. faba* plants infested by *H. halys* (Fig. 1). Over the observation period, all tested females made a response to volatiles. In particular, *H. halys* feeding and oviposition on the plant stimulated a marginally not significant response in the wasps compared to unexposed plants (*t* = −1.81; *df* = 29; *p* = 0.08). Instead, the wasps significantly preferred the volatiles released by unexposed plants, compared to volatiles from plants damaged by *H. halys* feeding activity (*t* = −2.42; *df* = 39; *p* = 0.02).

Response of *T. basalis* to volatiles induced by plants subjected to concurrent infestation of *H. halys* and *N. viridula.*

**Fig. 2:**
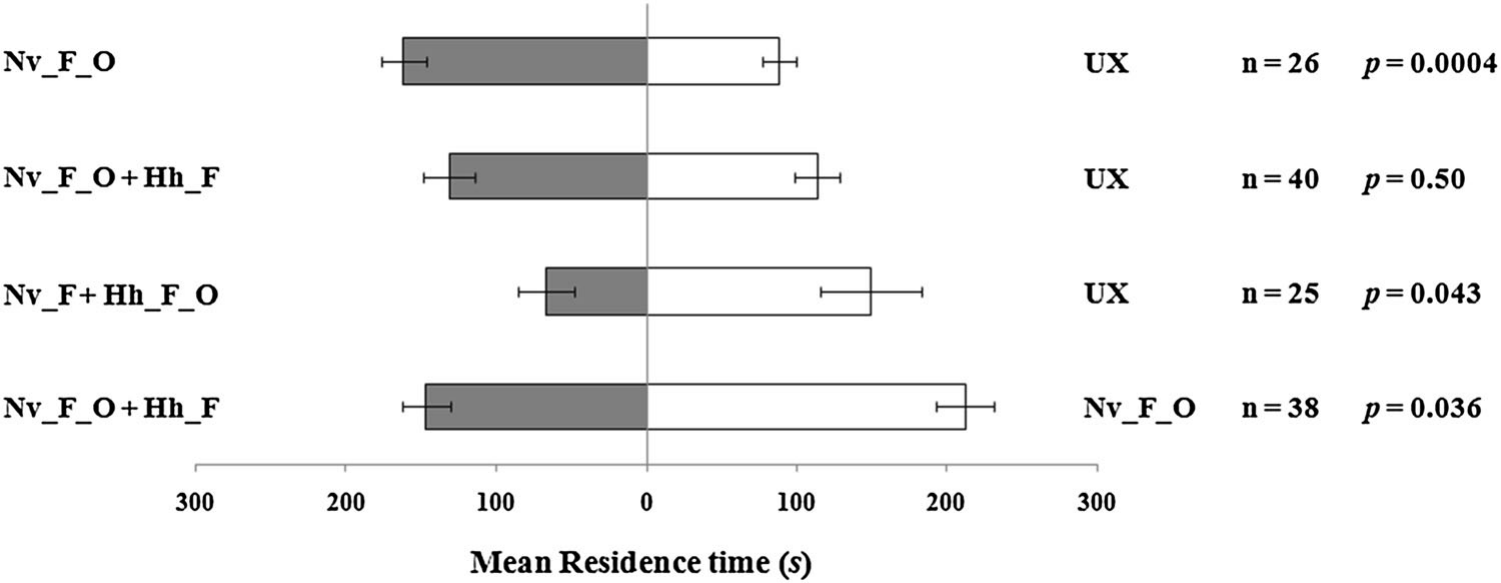
Response of *Trissolcus basalis* females to *Vicia faba* plant volatiles induced by concurrent infestation of *Halyomorpha halys* and *Nezara viridula.* Plant treatments: *N. viridula* feeding and oviposition (Nv_F_O); *N. viridula* feeding and oviposition and *H. halys* feeding (Nv_F_O + Hh_F); *N. viridula* feeding and *H. halys* feeding and oviposition (Nv_F + Hh_F_O); unexposed (UX). *Bars* represent mean (±SE) of the time spent by wasp females in each arm of the Y-tube olfactometer over an observation period of 300 s (paired *t* tests)

The response of *T. basalis* females to VOCs emitted by *V. faba* plants infested simultaneously by *H. halys* and *N. viridula* was affected by the plant treatments (Fig. 2). Over the observation period, all tested females made a response to volatiles. Wasps were significantly attracted to volatiles emitted by plants attacked by *N. viridula* feeding and oviposition compared to unexposed plants (*t* = 4.12; *df* = 25; *p* = 0.0004), whereas they were not attracted when plants were concurrently exposed to *N. viridula* feeding and oviposition activity and *H. halys* feeding, over unexposed plants (*t* = 0.68; *df* = 39; *p* = 0.50). Female wasps exhibited a significant preference for volatiles released by unexposed plants when tested versus plants concurrently damaged by *N. viridula* feeding and *H. halys* feeding and oviposition (*t* = −2.10; *df* = 24; *p* = 0.043). When volatiles from plants damaged by *N. viridula* feeding and oviposition were tested against plants exposed to concurrent infestation of *N. viridula* and *H. halys, T. basalis* females showed significant preference for plants attacked only by *N. viridula* (*t* = −2.17; *df* = 37; *p* = 0.036).

## Discussion

In the present study, we demonstrated that the alien herbivore *H. halys* can interfere with the local tritrophic system *V. faba*–*N. viridula*–*T. basalis*.

First, the attack of *H. halys* on *V. faba* plants modifies the response of *T. basalis* to VOCs emitted by uninfested plants. Indeed, the wasp females are not attracted by uninfested plants, but in the presence of plants damaged by *H. halys*, they choose constitutive *V. faba* VOCs, showing a preference that is statistically significant towards plants with *H. halys* feeding activity and marginally not significant towards plants with *H. halys* feeding and oviposition activities. Moreover, we detected that *T. basalis* females, which usually respond to plant volatiles induced by *N. viridula* feeding and oviposition (Colazza et al. 2004a, b), are not attracted by plants that were exposed to *H. halys* feeding and oviposition. The lack of response of *T. basalis* to OIPVs from *H. halys*-infested plants could be interpreted as a direct consequence of the absence of coevolution among the plant, alien herbivore and parasitoid. Plants have evolved adaptive indirect defence responses against coevolved herbivores, by recruiting natural enemies, but they could be more exposed when they have to deal with a novel non-coevolved herbivore (Desurmont et al. 2014). From a parasitoid point of view, this lack of attraction to plant infested by an alien and non-associated host herbivore may have beneficial or negative consequences in terms of its foraging efficiency, depending on its ability to successfully develop on alien host. Indeed, if the parasitoid is able to develop in the new host, its inability to use the new chemical cues provided by the plant–herbivore complex reduces the possibility to use a valuable host. In this case, the lack of attraction could be detrimental to the wasp fitness. On the contrary, when the parasitoids could not complete the development in the new host but they still respond to cues from the plant–herbivore complex, they might invest time and energy in unproductive foraging behaviour. In this respect, the alien herbivore may represent a sink or an ‘evolutionary trap’ for the natural enemies that undergo a reduction in their populations. Indirectly, this might be an advantage for local herbivore stink bugs, which might exhibit significant outbreaks due to a lower demographic pressure by natural enemies (Abram et al. 2014).

Our preliminary laboratory tests suggest that *H. halys* is a potential suitable host for *T. basalis*, even if its reproductive rate on *H. halys* fresh eggs is very low (about 10%, M.C. Foti personal observation), consistent with the results obtained by Haye et al. (2015), which observed a poor development of *Trissolcus* spp. on *H. halys* fresh eggs. Therefore, this host specificity could allow *T. basalis* to optimize time and energy by exploiting on cues emitted by suitable host species. This ability of *T. basalis* was already observed during orientation induced by substrate-borne kairomones. Indeed, foraging wasp females exploit more deeply chemical traces of the associated host, showing capacity to distinguish chemical traces of associated species from those of non-associated species (Salerno et al. 2006), and host sex discrimination ability only in the presence of chemical footprints from their associated host species (Peri et al. 2006, 2013).

Our data also show that herbivory by the alien stink bug *H. halys* disrupts *T. basalis* attraction towards OIPVs emitted by *V. faba* plants following attacks by *N. viridula*. The effect of multiple herbivory attacks on plant volatile emission and therefore on natural enemy recruitment is well reported in the literature, showing that it is a widespread ecological phenomenon, since it can occur not only when the attackers have similar feeding habits (results in this study; Shiojiri et al. 2001; Bukovinszky et al. 2012), but also when plants are under concurrent attacks by above and belowground herbivores (Rasmann and Turlings 2007; Soler et al. 2007), by piercing–sucking and chewing herbivores (Erb et al. 2010; Cusumano et al. 2015), by herbivorous insects and mites (de Boer et al. 2008; Zhang et al. 2009), and by insect herbivores and plant pathogens (Ponzio et al. 2014). Concerning alien insect herbivores, their impact on tritrophic interactions has been investigated in a few cases, and the results generally indicate that the attraction of parasitoids towards HIPVs and OIPVs is disrupted when both host and non-host herbivores were simultaneously present on the same plant (Desurmont et al. 2014; Chabaane et al. 2015; Clavijo McCormick 2016). For example, Cusumano et al. (2015) demonstrated that the attraction of the egg parasitoids *Trichogramma brassicae* Bezdenko and *T. evanescens* Westwood towards *Brassica nigra* L. is disrupted when the plants are under the attack of a naturally associated host, *Pieris brassicae* L., and by an alien and invasive herbivore, *Spodoptera exigua* (Hübner).

The effect exerted by *H. halys* in our tritrophic system not only provides an additional example of disturbance of an infochemical web caused by an invasive insect herbivores, but it also raises the doubt whether the *V. faba* plant–*T. basalis* signalling is a stable communication that can benefit the organisms involved. Indeed, the alteration reported here represents the third case for this plant–parasitoid interaction. Previous studies have shown that the *T. basalis* attraction to *V. faba* OIPVs, emitted after *N. viridula* egg deposition and feeding activity, is altered by biotic and abiotic stresses. Moujahed et al. (2014) demonstrated that the concurrent infestation of non-host beetle *S. lineatus*, either adults feeding on leaves or larvae feeding on roots, reduces the attraction of *T. basalis* towards OIPVs emitted by *V. faba* plants infested by *N. viridula*. On the contrary, the volatile blend emitted by *V. faba* plants that were under simultaneous water stress and *N. viridula* attack enhances the attraction of *T. basalis* (Salerno et al. 2017). However, to better understand the role of OIPVs in mediating broad bean plant–egg parasitoid interaction, the influence played by parasitoid learning should be considered. In fact, it has been suggested that egg parasitoids could rely on learning abilities when foraging for hosts in complex and dynamic environments (Fatouros et al. 2008; Colazza et al. 2010; Cusumano et al. 2012). Indeed, a partially adaptive learning was demonstrated in *T. basalis* females, which are attracted to volatiles induced in *V. faba* plants infested by a non-host herbivorous species (*S. lineatus* adults or larvae) when they are naïve, but not after an oviposition experience (Moujahed et al. 2014). Similarly, Cusumano et al. (2015) suggested that associative learning could be important in foraging behaviour of *Trichogramma* species, such as *T. evanescens* and *T. brassicae* when exploiting OIPVs emitted by *Brassica* plants under multiple herbivore attacks.

In summary, our laboratory study evidences the disruptive impact of an alien insect herbivore on plant volatile-mediated signalling in a local tritrophic web. However, further research on multitrophic interactions under field or semi-field conditions is required to better determine the impact of *H. halys* on *T. basalis* recruitment by infested plants and, consequently, on egg parasitoid efficacy in controlling pentatomid hosts.

## Author contribution statement

LM, MCF, EP and SC conceived and designed the research. MCF and LM conducted the experiments. MCF and EP analysed data. MCF, LM and EP wrote the manuscript. EP, SC, GR and EC reviewed the manuscript. All authors read and approved the manuscript.
